# Cost-Utility Analysis of Chuna Manual Therapy and Usual Care for Chronic Neck Pain: A Multicenter Pragmatic Randomized Controlled Trial

**DOI:** 10.3389/fmed.2022.896422

**Published:** 2022-05-11

**Authors:** In-Hyuk Ha, Eun-San Kim, Sook-Hyun Lee, Yoon Jae Lee, Hyun Jin Song, Younhee Kim, Koh-Woon Kim, Jae-Heung Cho, Jun-Hwan Lee, Byung-Cheul Shin, Jinho Lee, Joon-Shik Shin

**Affiliations:** ^1^Jaseng Spine and Joint Research Institute, Jaseng Medical Foundation, Seoul, South Korea; ^2^College of Pharmacy, University of Florida, Gainesville, FL, United States; ^3^College of Medicine, Inha University, Incheon, South Korea; ^4^Department of Korean Rehabilitation Medicine, College of Korean Medicine, Kyung Hee University, Seoul, South Korea; ^5^Clinical Research Division, Korea Institute of Oriental Medicine, Daejeon, South Korea; ^6^Korean Medicine Life Science, Campus of Korea Institute of Oriental Medicine, University of Science and Technology, Daejeon, South Korea; ^7^School of Korean Medicine, Pusan National University, Yangsan, South Korea; ^8^Spine & Joint Center, Pusan National University Korean Medicine Hospital, Ysangsan, South Korea; ^9^Jaseng Hospital of Korean Medicine, Seoul, South Korea

**Keywords:** chronic neck pain, cost-utility analysis, pragmatic randomized controlled trial, manual therapy, Chuna

## Abstract

This study aimed to compare the cost-effectiveness of manual therapy and usual care for patients with chronic neck pain. A cost-utility analysis alongside a pragmatic randomized controlled trial was conducted in five South Korean hospitals. Data were procured from surveys and nationally representative data. Participants were 108 patients aged between 19 and 60 years, with chronic neck pain persisting for at least 3 months and a pain intensity score of ≥5 on the numerical rating scale in the last 3 days. The study was conducted for 1 year, including 5 weeks of intervention and additional observational periods. Participants were divided into a manual therapy (Chuna) group and a usual care group, and quality-adjusted life-years, costs, and the incremental cost-effectiveness ratio were calculated. The quality-adjusted life-years of the manual therapy group were 0.024 higher than that of the usual care group. From the societal perspective, manual therapy incurred a lower cost—at $2,131—and was, therefore, the more cost-effective intervention. From a healthcare system perspective, the cost of manual therapy was higher, with an incremental cost-effectiveness ratio amount of $11,217. Manual therapy is more cost-effective for non-specific chronic neck pain management from both a healthcare system and societal perspective.

## Introduction

Neck pain is a major cause of functional disability worldwide. It leads to an economic burden due to increased societal costs from loss of productivity and healthcare system costs (1, 2). Particularly, the management of chronic neck pain has important socioeconomic implications because of the high incidence of neck pain among young and economically productive age groups (2).

Interventions for neck pain treatment have largely been based on conventional medicine, such as medication and physical therapy (3). However, in recent years, the use of complementary and alternative medicine for pain management has become common (4, 5). Specifically, recent guidelines have recommended non-pharmacologic interventions, such as acupuncture and manual therapy to treat musculoskeletal pain (6).

A systematic review in 2014 reported inconsistent findings on the cost-effectiveness of manual therapy for neck pain (7); one study demonstrated that manual therapy was more cost-effective than physical therapy or general practitioner care (8), while other studies have demonstrated that manual therapy was less effective than behavioral graded activity (9). Moreover, the probability of manual therapy being cost-effective was too low compared to the care with advice plus exercise (10).

Chuna manual therapy is a type of manual therapy that achieves therapeutic effects by aiming to create balance regarding the physiological and pathological conditions of the human body. The current modernized form of Chuna manual therapy was developed based on traditional Chuna techniques with a theoretical basis in Korean medicine (11). Chuna manual therapy for the treatment of musculoskeletal disorders has been covered by the South Korean national health insurance since April 2019. Subsequently, it became possible to examine the use of manual therapy for musculoskeletal disorders to determine its efficacy and cost-effectiveness (12, 13). A recent study demonstrated that Chuna manual therapy (Chuna) was more effective in relieving pain and improving function and quality of life than usual care (i.e., Western medicine, such as physical therapy) (11). However, little is known about the cost-effectiveness of Chuna among patients with chronic neck pain. Economic evaluations provide important data that guide healthcare providers and decision-makers in the allocation of resources in healthcare decision-making to optimize public health (14). Therefore, this study conducted the first cost-utility analysis alongside a pragmatic randomized controlled trial to compare Chuna with usual care for chronic neck pain from both healthcare system and societal perspectives.

## Materials and Methods

We conducted a multicenter pragmatic randomized clinical trial at five South Korean medical hospitals from 27 September 2017 to 28 June 2019. Written informed consent was obtained from the 108 patients recruited for this study. The institutional review board approved the trial protocol. The study commenced after registering the trial on the clinical trials registration site (ClinicalTrials.gov Identifier: NCT03294785). As the trial protocol and results have already been published (11, 15), its details can be found in the protocol paper; the content of the clinical trial is briefly described in this article. The Consolidated Health Economic Evaluation Reporting Standards Statement (CHEERS) checklist (16) is supplemented according to the guidelines (17).

### Eligibility Criteria and Interventions

Patients aged between 19 and 60 years, with chronic neck pain persisting for at least 3 months and a pain intensity score of ≥5 on the numerical rating scale (NRS) in the last 3 days were eligible for the study. The exclusion criteria were as follows: those (1) with severe neurological symptoms, such as progressive neurological defect, history of cervical spine surgery, or specific severe comorbidity that could cause chronic neck pain (e.g., tumor or fracture), (2) who used medications (e.g., psychiatric drugs, steroids, or immunosuppressants) that could interfere with the study results, or (3) who used medication or had received treatment within the last week that could affect pain, such as Chuna, physical therapy, or non-steroidal anti-inflammatory drugs.

Stratified block randomization was performed. Participants were randomly allocated to 2 groups, a Chuna group and a usual care group. The randomly generated treatment group allocations were delivered to each center in individually sealed opaque envelopes for allocation concealment. Both groups received two treatment sessions per week for five consecutive weeks, resulting in ten treatment sessions in total. The physicians selected the Chuna technique based on their clinical judgment by referring to “Chunauihak” (3.0rd Edition, Korean Society of Chuna Manual Therapy, “Chuna Medicine”) (18). Of the 43 types of Chuna manual therapy techniques, the systematic application techniques—including those for the cervical area—were employed without any limitation to the number of techniques used. The usual care group was provided with oral medication and physical therapy (electrotherapy and thermotherapy). To reflect the clinical environment in South Korea, we used national data from the 2014 Korean Health Insurance Review and Assessment Service (HIRA) Service-National Patient Sample database and extracted a list of the most frequently used drugs and physical therapy treatments for neck pain (19) and provided them to the medical personnel in charge of the usual care group for reference when prescribing medications. During the treatment period, apart from the interventions, only the rescue drug acetaminophen (up to 4 g/day) was allowed, and its dose was recorded. There were no restrictions on treatments after the primary endpoint (5 weeks from baseline), and all treatments were recorded.

### Utility Weights

Utility weights were assessed using the EuroQol 5-Dimension 5-Level scale (EQ-5D-5L) developed by the EuroQol Group. Scores were calculated using the mapping equation and validated using time-trade off in the South Korean general population (20). We used the EQ-5D-5L for a base-case scenario and the Short-Form 6-Dimensional health state (SF-6D). The SF-6D scores were calculated using the 12-Item Short-Form Health Survey version 2 (SF-12v2) according to Brazier and Roberts (21) Utility weights range from 0 (death) to 1 (perfect health), with a higher score indicating a better health status. Utilities were measured at the baseline and at 5, 13, 26, 39, and 52 weeks thereafter; the missing rates were at 0, 8, 5, 6, 6, and 6%, respectively. To calculate quality-adjusted life-years (QALYs) from utility weights, we used the trapezoidal rule method.

### Costs

We estimated medical and non-medical costs from a healthcare system perspective. Medical costs included formal and informal healthcare costs for chronic neck pain treatment, and non-medical costs included transportation and time (22). In addition, we estimated productivity costs to estimate total costs from a societal perspective. Administered Chuna, physical therapy, examination, and prescription drugs were recorded in detail in case report forms. The corresponding costs were calculated using the 2019 price index from HIRA.

We used the patients' survey data, obtained through the questionnaire, to calculate personal expenses (e.g., over-the-counter drugs). Additional private outpatient visits (i.e., Korean traditional medicine and Western medicine) and chronic neck pain management related to healthcare usage (e.g., aids, exercise, and massage) were recorded in the case report form. During the study period, the survey was conducted at 5, 13, 26, 39, and 52 weeks from the baseline, and the missing rates for each endpoint were 8, 5, 6, 6, and 6%, respectively. For additional private outpatient visits, only out-of-pocket payments for health insurance benefit services and non-benefit services were surveyed; therefore, South Korean national health insurance benefits were estimated using the HIRA—2018 National Patient Sample (23).

For non-medical costs, the transportation and time costs spent by the patient to receive Chuna and physical therapy were surveyed using a questionnaire. Time was then converted into cost through sex- and age-stratified income (24). To estimate productivity costs, the human capital approach was applied by multiplying the productivity loss with sex- and age-stratified incomes (24). The productivity loss due to chronic neck pain was measured using the Work Productivity and Activity Impairment: Specific Health Problem (WPAI-SHP) questionnaire (25), which measures absenteeism, presenteeism, and overall work impairment (combined index of absenteeism and presenteeism) due to specific health problems (i.e., chronic neck pain) in the past week for employed respondents. Activity impairment (impairment in regular activities) was measured for both employed and unemployed patients. WPAI-SHP was measured at 1–5 weeks, 13, 26, 39, and 52 weeks from baseline, and the missing rate was between 6 and 10%.

Our base-case analysis used overall work impairment for employed patients and activity impairment for unemployed patients to calculate productivity costs as ~30% of patients were unemployed; this ratio was maintained until the end of the follow-up. If productivity costs were estimated only for employed patients, the opportunity costs due to unpaid work could be underestimated (26). The result when estimating the productivity costs for only employed patients is presented in the sensitivity analysis section. Total costs per year for each intervention were estimated, as the study period was 1 year, and no discount rate was applied. All costs were converted to United States Dollar (USD, hereafter indicated as $) at the 2019 exchange rate (1,156 KRW = $1) (27). Costs estimated using external data sources were converted with reference to 2019 values according to the inflation rate. Details on the estimation method of the costs are described in Supplementary Appendix 1.

### Cost-Utility Analysis

The intention-to-treat analysis was conducted as the base-case analysis, and the missing values of cost and utility were filled in using multiple imputations. We included treatment allocation, sex, and age as baseline covariates to construct the imputation model. Other covariates were included in the imputation model according to their correlations (28). Due to the skewness in the data distribution, imputation was performed by predictive mean matching. A Markov chain Monte Carlo method was used for the estimation process, and a total of 20 imputation sets were generated. The mice package version 3.6.0 in R version 4.0.1 was used for missing data imputation.

For the cost-utility analysis, the incremental cost-effectiveness ratio (ICER) was calculated by dividing the difference in costs by the difference in utility between the two groups. As there were uncertainties from the distribution, 10,000 sample means were extracted through non-parametric bootstrapping. The cost-effectiveness plane was derived from the differences in the extracted cost and QALY, and the probability of the extracted sample being included in each quadrant of the plane was calculated. Next, the incremental net benefit and cost-effectiveness acceptability curves were obtained considering the “willingness to pay” (WTP; 30,050,000 KRW; $26,375), based on values determined by a previous study (29).

### Sensitivity Analysis

Three different scenarios of sensitivity analyses were performed:

(1) The complete case analysis was performed for 49 patients (91%) in the Chuna group and 40 patients (74%) in the usual care group.
(2) In the base-case analysis, we used productivity loss within the previous 1 week, as the WPAI-SHP was validated by surveying the work and activity impairment during the previous week. However, when the interval between visits was longer than 1 week (3, 6, 9, and 12 months from baseline), the productivity loss might not be accurately assessed due to the possibility of the patient having undergone accidental events. Therefore, at 3, 6, 9, and 12 months from the baseline, productivity loss from the last to the present visit was also surveyed using the framework of the WPAI-SHP questionnaire.
(3) The productivity costs were calculated by overall work impairment for employed patients only; therefore, the productivity costs for unemployed patients were set as 0.

### Patient and Public Involvement

No patients were involved in the design or implementation of this study, setting the research questions, or determining the outcome measures of this study, nor did they have any input on the data analysis, interpretation, or writing up of results. The trial results will be shared with all participants through the research paper and the Clinical Research Information Service (KCT0002732).

### Role of the Funding Source

The study sponsors were not involved in the study design, data collection, analysis, interpretation, manuscript writing, or the decision to submit the article for publication.

## Results

There were no statistical differences in the demographic and clinical characteristics, namely, EQ-5D-5L, SF-6D (i.e., utility weights), and WPAI-SHP (i.e., productivity loss), between the two groups at the baseline (Table 1). In the analysis of effectiveness between the two groups, the manual therapy group demonstrated superior results in terms of pain and function compared with the usual care group. In the survival analysis, where recovery was defined as a ≥50% decrease in neck pain on the NRS, the manual therapy group demonstrated a more rapid recovery rate (11). The number of patients who completed the evaluation of outcomes included in the cost-utility analysis at all-time points was 49 (91%) in the Chuna group and 40 (74%) in the usual care group.

**Table 1:** Demographic and clinical characteristics of the patients at baseline^*^.

	**Manual therapy**	**Usual care**	***P*** **-Value**
	**(*n* = 54)**	**(*n* = 54)**	
**Sex**			**1**
Female	36 (66.7)	37 (68.5)	
Male	18 (33.3)	17 (31.5)	
Age	39.3 (8.2)	37.5 (10.3)	0.307
Body mass index	23.2 (3.8)	23.0 (3.1)	0.749
Pain duration (months)	49.0 (43.4)	48.2 (40.5)	0.924
**Previous medical use** ^†^			**0.55**
Yes	18 (33.3)	22 (40.7)	
No	36 (66.7)	32 (59.3)	
**Radiating arm pain**			**0.335**
Yes	32 (59.3)	26 (48.1)	
No	22 (40.7)	28 (51.9)	
**Pain type (current)**			**0.404**
Continuous	29 (53.7)	33 (61.1)	
Fluctuating	25 (46.3)	20 (37.0)	
Unknown	0 (0.0)	1 (1.9)	
**Sensory deficiency**			**1**
Yes	5 (9.3)	6 (11.1)	
No	49 (90.7)	48 (88.9)	
**Motor weakness**			**0.713**
Yes	5 (9.3)	3 (5.6)	
No	49 (90.7)	51 (94.4)	
**Straight neck**			**0.817**
Yes	41 (75.9)	43 (79.6)	
No	13 (24.1)	11 (20.4)	
**Cervical disc space narrowing**			**0.417**
Yes	21 (38.9)	16 (29.6)	
No	33 (61.1)	38 (70.4)	
**Degeneration**			**0.817**
Yes	13 (24.1)	11 (20.4)	
No	41 (75.9)	43 (79.6)	
**VAS** ^§^			
Neck	59.5 (13.1)	60.6 (10.6)	0.619
Arm	33.3 (26.5)	28.1 (26.4)	0.307
**NRS** ^ǁ^			
Neck	5.9 (1.2)	6.2 (0.8)	0.145
Arm	3.3 (2.7)	2.9 (2.7)	0.394
NPQ^¶^	38.4 (12.9)	36.8 (11.5)	0.503
NDI^**^	33.0 (11.6)	32.3 (10.6)	0.741
EQ-5D-5L score^††^	0.76 (0.11)	0.77 (0.11)	0.547
**SF-12 score**			
Physical component summary	41.7 (6.1)	44.6 (6.5)	0.019
Mental component summary	49.3 (10.0)	48.1 (9.9)	0.505
SF-6D score^§§^	0.69 (0.12)	0.71 (0.11)	0.382
WPAI-SHP^ǁǁ^	53.1 (18.1)	49.0 (18.7)	0.249

*VAS, visual analog scale; NRS, numeric rating scale; NPQ, northwick park questionnaire; NDI, neck disability index; EQ-5D-5L, EuroQol 5-dimension 5-level; SF-12, 12-item short-form general survey; SF-6D, six-dimensional health state short form; WPAI-SHP, the work productivity, and activity impairment questionnaire: specific health problem*.

* *Data are represented as either mean ± SD or number (%). Values of continuous variables between the two groups were compared using independent t-tests, and values of categorical variables were compared using the chi-square test or Fisher's exact test*.

† *Any medical intervention used by the patient within the last 3 months to alleviate neck pain*.

*Diagnosed by a radiology consultant after X-ray imaging*.

§ *The VAS score of pain was measured by having patients indicate their pain level on a line, from 0 (no pain) to 100 (most severe pain imaginable), in millimeters*.

ǁ *The numeric rating scale score of pain was measured by having patients report their pain level as a number from 0 (no pain) to 10 (most severe pain imaginable)*.

¶ *The Northwick Park Questionnaire score was calculated as a percentage, where higher scores indicate more severe pain and disability*.

** *The Neck Disability Index score was calculated as a percentage, where higher scores indicate a more severe disability*.

†† *The EuroQol 5-Dimension 5-Level score was calculated by converting patient responses on a scale from−0.066 (lowest quality of life) to 1 (highest quality of life)*.

*The Medical Outcomes Study 12-Item Short-Form General Survey score was calculated by converting patient responses on a scale from 0 (lowest quality of life) to 100 (highest quality of life)*.

§§ *The six-dimensional health state short form was calculated using the method developed by Brazier and Roberts (21), and the score ranges from 0 (lowest quality of life) to 1 (highest quality of life)*.

ǁǁ *The Work Productivity and Activity Impairment Questionnaire: Specific Health Problem score was calculated as a percentage, evaluating the overall work impairment due to neck pain during the last week. For patients who were unemployed or not working in the last week, the activity impairment was rated*.

### QALYs

**Figure 1:**
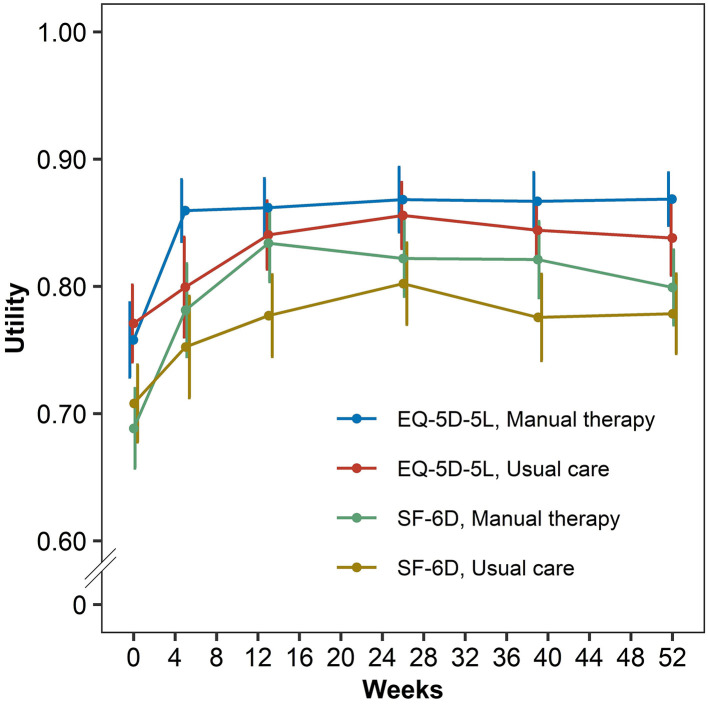
Distribution in utilities according to EQ-5D-5L and SF-6D by manual therapy and usual care.

For both the EQ-5D-5L and SF-6D, the Chuna results demonstrated a higher mean utility value than usual care at all-time points, except for the baseline (Figure 1). The difference in EQ-5D-5L score between the two groups was the greatest and statistically significant at the fifth week from the baseline (0.06; 95% CI 0.01–0.11), which is the end of the intervention period; as for the SF-6D, the difference was the greatest in the first quarter or 3 months from baseline (0.06; 95% CI 0.01–0.10). QALYs using EQ-5D-5L in the Chuna group and usual care group were 0.860 and 0.836, respectively, the Chuna group had incremental QALYs of 0.024, compared with the usual care group (95% CI 0.000–0.048). Similarly, the QALY calculated using SF-6D was 0.810 in the Chuna group and 0.777 in the usual care group, with a difference of 0.033 (95% CI 0.001–0.065; Table 2).

**Table 2:** Quality-adjusted life-years (QALY) by manual therapy and usual care^*^.

	**Manual therapy**	**Usual care**	**Difference**	***P*** **-Value**
	**(*n* = 54)**	**(*n* = 54)**		
**EQ-5D-5L**				
Baseline to 5th week	0.86 (0.84 to 0.88)	0.80 (0.76 to 0.84)	0.06 (0.01 to 0.11)^*^	0.013
1st quarter	0.86 (0.84 to 0.88)	0.84 (0.81 to 0.87)	0.02 (−0.01 to 0.06)	0.238
2nd quarter	0.87 (0.84 to 0.89)	0.86 (0.83 to 0.88)	0.01 (−0.02 to 0.05)	0.502
3rd quarter	0.87 (0.84 to 0.89)	0.84 (0.82 to 0.87)	0.02 (−0.01 to 0.05)	0.163
4th quarter	0.87 (0.85 to 0.89)	0.84 (0.81 to 0.87)	0.03 (−0.01 to 0.07)	0.097
QALYs	0.860 (0.844 to 0.876)	0.836 (0.819 to 0.854)	0.024 (0.000 to 0.048)	0.052
**SF-6D**				
Baseline to 5th week	0.78 (0.74 to 0.82)	0.75 (0.71 to 0.79)	0.03 (−0.03 to 0.09)	0.310
1st quarter	0.83 (0.80 to 0.86)	0.78 (0.74 to 0.81)	0.06 (0.01 to 0.10)^*^	0.013
2nd quarter	0.82 (0.79 to 0.85)	0.80 (0.77 to 0.83)	0.02 (−0.02 to 0.06)	0.377
3rd quarter	0.82 (0.79 to 0.85)	0.78 (0.74 to 0.81)	0.05 (0.00 to 0.09)	0.051
4th quarter	0.80 (0.77 to 0.83)	0.78 (0.75 to 0.81)	0.02 (−0.02 to 0.06)	0.352
QALYs	0.810 (0.788 to 0.832)	0.777 (0.754 to 0.800)	0.033 (0.001 to 0.065)^*^	0.043

*QALY, quality-adjusted life-years; EQ-5D-5L, EuroQol 5-dimension 5-level; SF-6D, short form 6-dimensional health state*.

* *The QALYs were calculated using the trapezoidal rule. QALYs calculated in EQ-5D-5L were used for the base-case analysis, and SF-6D was used for the additional analysis. The 1st quarter to 4th quarter indicates from the baseline to 3, 3–6, 6–9, and 9–12 months, respectively. All values are presented by the mean and the 95% CI. The difference between the two groups was estimated using the independent t-test*.

^*^P < 0.05.

### Costs

The total medical costs of the Chuna group were higher than the usual care group ($213; 95% CI –$54–$480), and the period of the largest difference between the two groups was at 5 weeks from the baseline, which is the end of the intervention period. From a healthcare system perspective, the total costs (i.e., the sum of medical and non-medical costs) of Chuna were higher than usual care at $267 (95% CI –$11–$545). However, the productivity costs of Chuna had lower costs than usual care at all follow-up visits, and the difference in total productivity costs was –$2,398 (95% CI –$4,859–$63). Therefore, although Chuna had higher total medical and non-medical costs, the productivity costs were low. The difference in the total societal costs was -$2,131 (95% CI –$4,737–$475), indicating that the costs for Chuna were lower (Table 3). Details of the healthcare resources used and the associated costs are presented in the Supplementary Appendix.

**Table 3:** Costs per patient by manual therapy and usual care^*^.

	**Manual therapy**	**Usual care**	**Difference**	***P*** **-Value**
	**(*n* = 54)**	**(*n* = 54)**		
**Total medical costs**				
Baseline to 5th week	425 (420 to 431)	163 (147 to 179)	262 (245 to 279)^***^	<0.001
1st quarter	451 (424 to 478)	191 (168 to 215)	260 (223 to 296)^***^	<0.001
2nd quarter	51 (−9 to 110)	79 (23 to 134)	−28 (−111 to 54)	0.499
3rd quarter	109 (−5 to 222)	64 (14 to 114)	45 (−81 to 170)	0.482
4th quarter	61 (17 to 105)	124 (17 to 231)	−63 (−179 to 53)	0.283
Total	671 (483 to 858)	458 (272 to 643)	213 (−54 to 480)	0.116
**Non-Medical costs**				
Transportation	18 (7 to 28)	13 (6 to 19)	5 (−8 to 18)	0.435
Time loss for intervention	321 (278 to 364)	272 (227 to 317)	49 (−14 to 112)	0.124
Total	338 (293 to 383)	284 (237 to 331)	54 (−11 to 120)	0.105
**Healthcare system perspectives**				
Total	1,009 (817 to 1,202)	742 (545 to 939)	267 (−11 to 545)	0.06
**Productivity costs**				
Baseline to 5th week	1,386 (1,227 to 1,545)	1,602 (1,441 to 1,763)	−216 (−444 to 12)	0.063
1st quarter	3,471 (3,023 to 3,919)	3,984 (3,566 to 4,401)	−512 (−1,132 to 108)	0.104
2nd quarter	2,878 (2,347 to 3,410)	3,503 (3,002 to 4,005)	−625 (−1,365 to 115)	0.097
3rd quarter	2,925 (2,380 to 3,471)	3,360 (2,818 to 3,902)	−435 (−1,213 to 343)	0.27
4th quarter	2,660 (2,167 to 3,154)	3,487 (2,888 to 4,085)	−826 (−1,613 to −39)^*^	0.04
Total	11,935 (10,181 to 13,690)	14,333 (12,650 to 16,016)	−2,398 (−4,859 to 63)	0.056
**Societal perspectives**				
Baseline to 5th week	2,150 (1,978 to 2,322)	2,049 (1,870 to 2,229)	100 (−152 to 352)	0.433
1st quarter	4,261 (3,801 to 4,720)	4,459 (4,031 to 4,887)	−198 (−835 to 438)	0.537
2nd quarter	2,929 (2,368 to 3,490)	3,582 (3,066 to 4,099)	−653 (−1,426 to 119)	0.097
3rd quarter	3,034 (2,428 to 3,639)	3,424 (2,866 to 3,981)	−390 (−1,223 to 443)	0.355
4th quarter	2,721 (2,207 to 3,235)	3,610 (2,964 to 4,257)	−889 (−1,725 to −53)^*^	0.037
Total	12,944 (11,070 to 14,818)	15,075 (13,308 to 16,842)	−2,131 (−4,737 to 475)	0.108

* *Intervention period refers to the period from the baseline to the 5th week during which the manual therapy and usual care were provided. The 1st quarter to 4th quarter indicates from the baseline to 3, 3–6, 6–9, and 9–12 months, respectively. All values are presented by the mean and the 95% CI. The difference between the two groups was estimated using an independent t-test. KRW (Korea Won) was converted to USD (United States Dollar); 1 USD was calculated at 1,156 KRW*.

^*^ *P < 0.05;*

*** *P < 0.001*.

### Cost-Utility Analysis

From a societal perspective, Chuna had higher QALYs—calculated by EQ-5D-5L and SF-6D—and lower costs than usual care; therefore, Chuna was a better option. The cost-effectiveness acceptability curves demonstrated that the probability of cost-effectiveness of Chuna was more than 97% when WTP was $26,375 per QALY, which has been estimated as the threshold in the South Korean general population (29).

**Figure 2:**
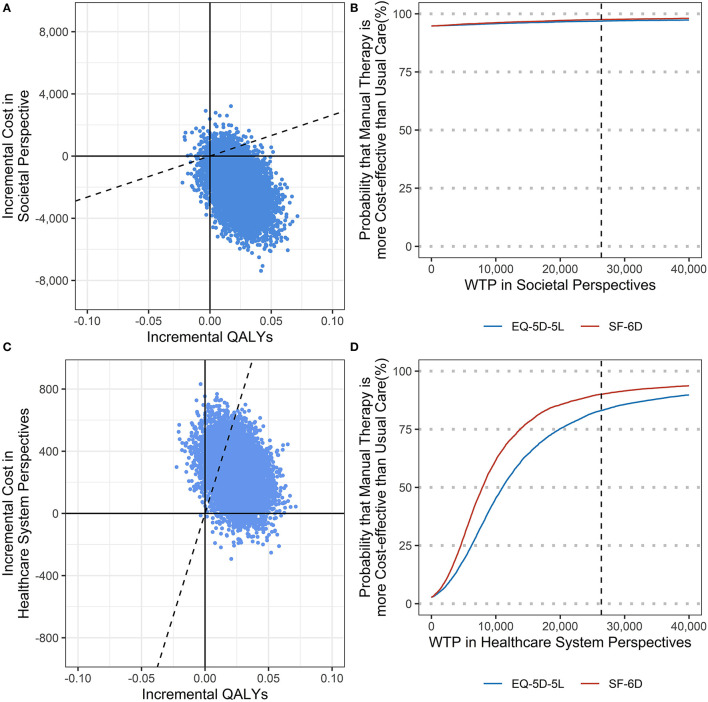
**(A)** Cost effectiveness plane in societal perspectives; **(B)** Cost effectiveness acceptability curve in societal perspectives; **(C)** Cost effectiveness plane in healthcare system perspectives; **(D)** Cost effectiveness acceptability curve in healthcare system perspectives.

From a healthcare system perspective, when compared with the usual care group, Chuna had incremental costs of $267 and incremental QALYs of 0.06 and 0.03, using EQ-5D-5L and SF-6D, respectively, thus, the ICER calculated by EQ-5D-5L and SF-6D was $11,217/QALY and $8,080/QALY, respectively (Table 4). The probability of Chuna being cost-effective was 83% and 90% in cases using EQ-5D-5L and SF-6D, respectively, compared to usual care (Figure 2; Supplementary Appendix 3). Regarding the sensitivity analyses, in Scenario 1, the complete case analysis, and Scenario 2, in which productivity loss was surveyed from the last visit to the present, the ICER and probability of cost-effectiveness were not significantly different from the base-case analysis. However, in Scenario 3, where productivity costs were considered zero for unemployed patients, the probability of cost-effectiveness of the societal perspectives decreased to 66%.

**Table 4:** The results of cost-effectiveness analysis for manual therapy compared with usual care^*^.

**QALY index**	**Main analysis**			
	**Societal perspectives**		**Healthcare system perspectives**	
	**EQ-5D-5L**	**SF-6D**	**EQ-5D-5L**	**SF-6D**
Difference in QALY	0.024 (0.000 to 0.048)	0.033 (0.001 to 0.065)^*^	0.024 (0.000 to 0.048)	0.033 (0.001 to 0.065)^*^
Difference in cost	−2,131 (−4,737 to 475)		267 (−11 to 545)	
ICER ($)	Dominant	Dominant	11,217	8,080
**Probability of cost-effectiveness by cost-effectiveness plane (%)**				
Cost-Saving + More effective	93	93	3	3
Cost-Increasing + More effective	5	5	95	95
Cost-Saving + Less effective	2	2	0	0
Cost-Increasing + Less effective	1	0	3	2
Probability of cost effectiveness at 1xWTP per capita (%)	97	98	83	90
Incremental net benefit at 1xWTP per capita ($)	2,778 (−178 to 5,572)	3,024 (7 to 5,908)	365 (−395 to 1,123)	609 (−330 to 1,540)

*QALY, quality-adjusted life-years; ICER, incremental cost-effectiveness ratio; EQ-5D-5L, EuroQol 5-dimension 5-level; SF-6D, the short form 6-dimensional health state*.

* For the base-case analysis, the QALY was calculated with EQ-5D-5L. The incremental cost is divided by the incremental QALY to calculate the incremental cost-effectiveness ratio (ICER). After nonparametric bootstrapping, the incremental net benefit (INB) and probability of cost-effectiveness were calculated using the 1xWTP threshold ($26,375). The costs from the healthcare system perspective include the costs of formal and informal healthcare involved in chronic neck pain treatment and the transportation and time costs. For the costs from the societal perspective, productivity costs from chronic neck pain were included. ^*^P < 0.05.

## Discussion

This study compared the intervention strategies of manual therapy and usual care for patients with chronic neck pain. The results of the analyses in this study demonstrate that Chuna is more cost-effective than usual care in terms of both the healthcare system and societal perspectives.

Although several clinical trials have conducted cost-effectiveness evaluations of Chuna in treating chronic neck pain, only a few studies could be compared with our study, considering the comparability of the disease and control group. We examined a 2014 systematic review on the cost-effectiveness of manual therapy for musculoskeletal conditions (7). There have been three studies on neck pain, two of which included patients with non-chronic neck pain, and only Lewis et al. (9) included a significant number of patients with chronic neck pain. They compared manual therapy with pulsed shortwave diathermy treatment, and both treatment options included advice and exercises. The participants in the manual therapy group had lower QALYs for 6 months than those in the pulsed shortwave diathermy group (0.342 vs. 0.360). The manual therapy used in this study was different from this previous study (9) in that it was performed by physicians, not physiotherapists. Also, the follow-up duration of this study was 1 year, while that of Lewis et al. (9) was 6 months, and this may have led to the difference in QALY between the two studies. In addition, healthcare and societal costs were slightly lower in the manual therapy group, and these results are considerably different from those of our study. Examining existing studies conducted after 2014, another study by Van Dongen et al. (30) compared manual therapy with physical therapy and drew no clear conclusions. However, manual therapy was also included with physical therapy, and therefore, essentially, it compared the two manual therapies. Moreover, Leininger et al. (31) compared spinal manipulative therapy with supervised exercise and home exercise to manage chronic neck pain and concluded that spinal manipulative therapy was a cost-effective option. Furthermore, Pach et al. (32) compared Chinese manual therapy (Tuina) and the No-Intervention Waiting List for chronic neck pain; the costs per QALY gained (ICER) in the Tuina group were calculated to be between €7,566 (€10.28 per session) and €39,414 (€35 per session).

The strengths of this study include the multicenter pragmatic clinical trial study design, reflecting the real-world clinical practice using national claims data. Specifically, to set the usual care for the control group, the treatment status of neck pain-related diseases in clinical practice was analyzed from the extracted national health insurance data (19). Moreover, the results of the analysis were provided for reference to the physicians responsible for deciding the type of treatment to provide for the control group in this study (11). Another strength lies in analyzing presenteeism using WPAI-SHP in estimating productivity loss due to chronic neck pain. Various studies have performed economic evaluations of manual therapy for chronic neck pain from a societal perspective but have analyzed only absenteeism (9, 31). However, when considering the characteristics of neck pain, it is important to investigate presenteeism and absenteeism. A study involving 10,000 Japanese workers reported that (33) neck pain or stiff shoulders ranked as the leading cause of productivity loss, and the annualized cost of presenteeism per capita was $414.05. In addition, in the United States, the estimated annual cost of productivity loss due to the presenteeism of neck pain per person was $1,690 (34). However, in this study, absenteeism due to chronic neck pain was rarely observed (data not shown).

Our study also presented some limitations which could be explored in future research. First, in the survey of the costs incurred during 1 year, most of the costs were self-reported, which may have introduced recall bias. Second, the Chuna group had a higher complete case rate (91%) than the usual care group (74%). Assuming that treatment preference affected the difference in the distribution of missing values, there may be concerns about the superior results of the Chuna group. Third, the employment rate considerably impacted the costs from a societal perspective. At the baseline, the difference in the employment rate between the two groups was only 3.7%, but in one follow-up period, the difference reached 16.8% (Supplementary Appendix 4). Therefore, in the sensitivity analysis in which the productivity costs were regarded as 0 when the patient was not a paid employee, the probability of cost-effectiveness had the lowest value (66%) compared with the results of other sensitivity analyses. This difference in employment rate may have been caused by the effect of the neck pain improvement from Chuna; however, the result should be interpreted with caution.

The subjects of this study were patients in their late 30s, with average pain duration of ~4 years, and although approximately 50% of these participants had the comorbidity of radiating arm pain, the degree of neck pain was not severe. In addition, it is thought that they were chronic neck pain patients of considerable severity with cervical disk space narrowing. More than half of these patients had continuous pain, but less than half indicated previous medical use in the last 3 months. It is possible that this result does not indicate that those patients did not require treatment but that they were either giving up on the treatment because they experienced no improvement with the usual care or had slow improvement even though they were undergoing treatment. Therefore, these characteristics of the patients may be the main reason the health utility value evaluation of manual therapy was significantly higher than that of the usual care. However, as usual, care is a much more commonly applied treatment method in South Korea, it is expected that Chuna and other forms of manual therapy can serve as an excellent alternative for numerous patients who do not show improvement or are unsatisfied with their existing treatment.

## Conclusion

This study demonstrated that manual therapy is cost-effective among patients with non-specific chronic neck pain, compared to usual care, from both societal and healthcare system perspectives. From a healthcare system perspective, the costs of manual therapy were higher than those of usual care. However, Chuna improved the participants' QALY scores, and the costs were acceptable. From a societal perspective, the productivity costs of Chuna were lower; therefore, the overall costs of Chuna were lower than that of usual care, indicating that Chuna was a better option. In conclusion, Chuna treatment for non-specific chronic neck pain is cost-effective in South Korea.

## Data Availability Statement

The original contributions presented in the study are included in the article/Supplementary Material, further inquiries can be directed to the corresponding author/s.

## Ethics Statement

The studies involving human participants were reviewed and approved by Institutional Review Board of Jaseng Hospital of Korean Medicine, Seoul, Korea. Written informed consent for participation was not required for this study in accordance with the national legislation and the institutional requirements.

## Author Contributions

I-HH, YL, JL, and J-SS: conceptualization. I-HH, E-SK, and YL: data curation, formal analysis, and methodology. I-HH: funding acquisition and writing—original draft. I-HH and YL: investigation and supervision. I-HH and S-HL: project administration. E-SK, S-HL, YL, HS, YK, K-WK, J-HC, J-HL, B-CS, JL, and J-SS: writing—review and editing. All authors contributed to the article and approved the submitted version.

## Funding

This research was supported by a grant from the Traditional Korea Medicine R&D Program through the Korea Health Industry Development Institute, funded by the Ministry of Health and Welfare, Republic of Korea (Grant No. HB16C0035).

## Conflict of Interest

The authors declare that the research was conducted in the absence of any commercial or financial relationships that could be construed as a potential conflict of interest.

## Publisher's Note

All claims expressed in this article are solely those of the authors and do not necessarily represent those of their affiliated organizations, or those of the publisher, the editors and the reviewers. Any product that may be evaluated in this article, or claim that may be made by its manufacturer, is not guaranteed or endorsed by the publisher.
