# Supplementary material for: Cost-Utility Analysis of Chuna Manual Therapy and Usual Care for Chronic Neck Pain: A Multicenter Pragmatic Randomized Controlled Trial
Source: Front Med (Lausanne). 2022 May 11;9:896422. doi: 10.3389/fmed.2022.896422 (PMC9131099; doi:10.3389/fmed.2022.896422)
Supplement: Supplementary file 1 [file Data_Sheet_1.docx]

**Supplementary Appendix 1.** Cost calculation method, associated data source, and unit cost*

| **Type of costs** | **Calculation method** | **Source of the Original Data** | **Unit cost** |
| --- | --- | --- | --- |
| Manual therapy (Chuna) † | The calculation was based on the pricing criteria of the Health Insurance Review and Assessment Service (HIRA). Chuna manual therapy is divided into simple, complex, and special according to the techniques applied. If there is a correction technique during the procedure, it is classified as complex, and during the intervention period, there was a correction technique in all procedures. | HIRA price index 2019 | $33 |
| Physical therapy  (Electrotherapy and thermotherapy) † | All details of the physical therapy performed on the patient are recorded, and the prices of the techniques corresponding to the HIRA price index are applied. | HIRA price index 2019 | $6  [4–11] |
| Consultation fee at the first visit by Korean medicine doctor | When a patient visits a medical institution, a medical doctor conducts an examination. The consultation fee is set differently for first and recursive visits, and it also differs between Korean medicine doctors and general practitioners. The manual therapy (Chuna) group was consulted by a Korean medicine doctor and the usual care group by a general practitioner. | HIRA price index 2019 | $12 |
| Consultation fee at recursive visits by Korean medicine doctor‡ |  | HIRA price index 2019 | $8 |
| Consultation fee at the first visit by western medicine doctor‡ |  | HIRA price index 2019 | $15 |
| Consultation fee at recursive visits by western medicine doctor‡ |  | HIRA price index 2019 | $11 |
| Syndrome differentiation technique fee‡ | Korean traditional medicine has a system that examines comprehensive symptoms based on unique theories and experiences. This is called syndrome differentiation (辨證), and all Korean medicine doctors may charge an additional syndrome differentiation technique fee (辨證技術料) according to the examination. This can be charged once a week, and the patients visited up to twice a week during the five-week intervention period. | HIRA price index 2019 | $3 |
| X-ray† | On the patient’s first visit, an X-ray examination of the cervical area was performed twice. | HIRA price index 2019 | $8 |
| Prescription§ | Through EMR, the type and dose of the drugs and the number of days of prescription drugs were surveyed during the intervention period. In the case of prescription drugs, all the costs were set by HIRA and were applied for each type of prescription drug. Dispensing fees are set by HIRA and were applied according to the number of days of prescription. | HIRA price index 2019 | $9  [4–13] |
| Over the counter¶ | If a patient purchases a prescription drug over the counter, the amount spent is surveyed. | Patient survey | $3  [2–3] |
| Additional private Korean traditional medicine outpatient visits** | The patient’s visits to medical institutions other than clinical trial sites and the associated expenses were surveyed. In this case, the amount indicated in the patient’s response was out-of-pocket payments for health insurance benefit services and non-benefit services, and the benefits were unknown. Accordingly, the benefits were calculated from the claim data of chronic neck pain patients in the Health Insurance Review and Assessment Service - National Patient Sample (HIRA-NPS) in 2018. The calculated average benefits were stratified by the patient’s sex and age and matched. | Patient survey  2018 HIRA NPS ^21^ | $54  [20–558] |
| Additional private Western medicine outpatient visits** |  |  | $111  [24–472] |
| Exercise, massage, etc.†† | The cost and number of services purchased by the patient were surveyed. | Patient survey | $24  [0–865] |
| Transportation‡‡ | The transportation cost to visit the clinical trial site was surveyed one week after the baseline, and this was multiplied by the number of visits. | Patient survey | $4  [1–24] |
| Time cost§§ | After one week from the baseline, all the time taken for the patient to leave the house, go to the hospital, interview, wait, treatment, and return home was surveyed. This was multiplied by the number of visits. | Patient survey | — |
| Productivity cost§§ | Productivity loss was assessed using Work Productivity and Activity Impairment – Specific Health Problem (WPAI-SHP). Then, according to the human capital approach, productivity loss was multiplied by sex and age-stratified income to calculate the income loss due to the productivity loss, which is regarded as the productivity costs. In the base case analysis, overall work impairment was applied to the productivity loss of employed patients, and activity impairment was applied to unemployed patients. The result of estimating the productivity costs only for employed patients is presented in the sensitivity analysis. | Patient survey | — |
| Income§§ | Sex- and age-stratified income was applied. | 2019 survey report on labor conditions by employment type ^22^ | — |

*Notes.* HIRA, Health Insurance Review and Assessment Service; HIRA-NPS, Health Insurance Review and Assessment Service - National Patient Sample; WPAI-SHP, Work Productivity and Activity Impairment – Specific Health Problem.

* In the case of applying a uniform cost, only the unit cost is presented. If costs are set in a patient-specific manner, the mean [min–max] of the calculated costs is indicated. All costs were converted to 2019 USD (1,156 KRW = 1 USD) (25).

† Price for each examination/treatment session

‡ Price for each visit to the clinical trial institution

§ Price for each prescription

¶ Price for each OTC purchase

** Price for each visit to the medical institution

†† Price for the one-time use of the service

‡‡ Price for each visit to the clinical trial institution

§§ For these costs, the costs incurred by the patients were not directly surveyed, and associated costs were calculated indirectly by considering the patients’ income. Therefore, the unit costs are not presented in these cases.

| **Supplementary Appendix 2.** Total healthcare costs and resources for manual therapy and usual care* | | | | |
| --- | --- | --- | --- | --- |
|  | **Manual therapy (n=54)** | | **Usual care (n=54)** | |
|  | **Volume**  **n (%)** | **Cost**  **Mean ± SD** | **Volume**  **n (%)** | **Cost**  **Mean ± SD** |
| **Manual therapy** (54/54) | 54 (100.0) | 318 ± 15 | — | — |
| **Physical Therapy** (54/54) | — | — | 54 (100.0) | 48 ± 27 |
| **Examination** (54/54) | 54 (100.0) | 103 ± 4 | 54 (100.0) | 105 ± 34 |
| **Prescriptions** (54/54) | 1 (1.9) | 0 ± 2 | 7 (13.0) | 4 ± 13 |
| **Travel cost** (52/46) | — | 18 ± 38 | — | 14 ± 26 |
| **Time cost** (52/46)† | 1,013 ± 472 | 323 ± 159 | 1,060 ± 522 | 305 ± 148 |
| **Total numbers of interventions received** | 9.8 ± 0.8 | — | 8.4 ± 3.1 | — |
| **Additional private healthcare usage** | | | | |
| **OTC** |  |  |  |  |
| Baseline to 5th week (54/54) | 1 (1.9) | 0 ± 1 | 0 (0.0) | 0 ± 0 |
| Total (54/49) | 4 (7.4) | 1 ± 3 | 1 (2.0) | 0 ± 1 |
| **Additional private Korean Traditional Medicine outpatient visits** | | | | |
| Baseline to 5th week (53/46) | 0 (0.0) | 0 ± 0 | 0 (0.0) | 0 ± 0 |
| 1st quarter (54/49) | 3 (5.6) | 5 ± 25 | 2 (4.1) | 2 ± 9 |
| 2nd quarter (53/48) | 0 (0.0) | 0 ± 0 | 3 (6.2) | 7 ± 33 |
| 3rd quarter (53/49) | 2 (3.8) | 8 ± 39 | 3 (6.1) | 11 ± 57 |
| 4th quarter (53/49) | 3 (5.7) | 20 ± 126 | 5 (10.2) | 32 ± 163 |
| Total (54/49) | 6 (11.1) | 31 ± 152 | 8 (16.3) | 52 ± 246 |
| **Additional private Western medicine outpatient visits** | | | | |
| Baseline to 5th week (53/46) | 0 (0.0) | 0 ± 0 | 1 (2.2) | 2 ± 16 |
| 1st quarter (54/49) | 6 (11.1) | 18 ± 96 | 4 (8.2) | 5 ± 20 |
| 2nd quarter (53/48) | 3 (5.7) | 28 ± 191 | 2 (4.2) | 19 ± 115 |
| 3rd quarter (53/49) | 4 (7.5) | 61 ± 398 | 3 (6.1) | 23 ± 111 |
| 4th quarter (53/49) | 3 (5.7) | 7 ± 35 | 5 (10.2) | 67 ± 251 |
| Total (54/49) | 8 (14.8) | 111 ± 624 | 11 (22.4) | 113 ± 431 |
| **Exercise** | | | | |
| Total (54/49) | 8 (14.8) | 31 ± 92 | 10 (20.4) | 54 ± 130 |
| **Massage** | | | | |
| Total (54/49) | 9 (16.7) | 50 ± 172 | 8 (16.3) | 74 ± 217 |
| **Etc.** | | | | |
| Total (54/49) | 14 (25.9) | 19 ± 65 | 11 (22.4) | 3 ± 8 |

*Notes*. * The 1^st^ quarter to 4^th^ quarter indicate the baseline to 3 months, 3 months to 6 months, 6 months to 9 months, and 9 months to 12 months, respectively. At each time point, the number of patients measured at least once during the period in the manual therapy and manual care groups is indicated. The number of patients that used the resources is indicated by n and percentage unless otherwise stated. Prices are expressed as mean ± SD KRW (Korean Won), and they were converted to USD (United States Dollar); 1 USD was calculated at 1,156 KRW.

† The unit is in “minute.”

**Supplementary Appendix 3.** Sensitivity analysis with cost-effectiveness analysis for manual therapy compared to usual care*

| **QALY index** | **Sensitivity analysis 1†** | | **Sensitivity analysis 2‡** | **Sensitivity analysis 3§** | |
| --- | --- | --- | --- | --- | --- |
|  | **Societal Perspectives** | **Healthcare System Perspectives** | **Societal Perspectives** | **Societal Perspectives** | **Healthcare System Perspectives** |
|  | **EQ-5D-5L** | | **EQ-5D-5L** | **EQ-5D-5L** | |
| Difference in QALY | 0.022 (-0.004 to 0.049) | | 0.024 (0.000 to 0.048) | 0.024 (0.000 to 0.048) | |
| Difference in cost | -2,110 (-5,000 to 780) | 229 (-95 to 554) | -2,271 (-4,853 to 311) | -71 (-3,425 to 3,284) | 230 (-54 to 514) |
| **ICER ($)** | Dominant | 10,289 | Dominant | Dominant | 9,660 |
| **Probability of cost-effectiveness by cost-effectiveness plane (%)** | | |  |  |  |
| Cost-saving + More effective | 90 | 8 | 94 | 51 | 5 |
| Cost-increasing + More effective | 6 | 88 | 3 | 47 | 93 |
| Cost-saving + Less effective | 3 | 0 | 2 | 1 | 0 |
| Cost-increasing + Less effective | 1 | 4 | 0 | 2 | 2 |
| **Probability of cost-effectiveness at 1xWTP per capita (%)** | 95 | 81 | 98 | 66 | 85 |
| **Incremental net benefit at 1xWTP per capita ($)** | 2,751 (-390 to 5,803) | 367 (-464 to 1,186) | 2,932 (74 to 5,692) | 697 (-2,796 to 4,212) | 411 (-354 to 1,146) |

Note. QALY, Quality-adjusted life-years; EQ-5D-5L, EuroQol 5-Dimension 5-Level; ICER, incremental cost-effectiveness ratio.

* For the base case analysis, the QALY was calculated with EQ-5D-5L. The incremental cost was divided by the incremental QALY to calculate the incremental cost-effectiveness ratio (ICER). After non-parametric bootstrapping, the incremental net benefit (INB) and probability of cost-effectiveness were calculated using the 1xWTP threshold ($26,375). The costs from the healthcare system perspective include the costs of formal and informal healthcare involved in chronic neck pain treatment and the transportation and time costs. For the costs in the societal perspective, productivity costs from chronic neck pain are included. * P < 0.05.

†Sensitivity analysis 1. Complete case analysis was performed. The 49 patients in the manual therapy group and 40 patients in the usual care group were included.

‡Sensitivity analysis 2. The WPAI was validated by surveying the work and activity impairment during the last week. However, when the interval between visits was longer than one week (3, 6, 9, and 12 months from baseline), the productivity loss might not be accurately assessed because of the possibility of the patient having undergone accidental events. Therefore, at 3, 6, 9, and 12 months from the baseline, productivity loss from the last to the present visit was additionally surveyed using the WPAI-SHP questionnaire framework.

§Sensitivity analysis 3. The productivity costs for unemployed patients were regarded as zero.

**Supplementary Appendix 4.** Distribution of the employment rates by manual therapy and usual care according to each measurement time point*

|  | **Manual therapy (%)** | **Usual care (%)** | **Difference (%)** |
| --- | --- | --- | --- |
| Baseline | 68.5 | 64.8 | 3.7 |
| 1st week | 69.3 | 61.2 | 8.1 |
| 2nd week | 71.9 | 59.8 | 12.1 |
| 3rd week | 72.2 | 59.4 | 12.8 |
| 4th week | 73.9 | 57.8 | 16.1 |
| 5th week | 74.1 | 61.0 | 13.1 |
| 3rd month | 77.8 | 62.1 | 15.6 |
| 6th month | 79.2 | 62.4 | 16.8 |
| 9th month | 70.3 | 66.0 | 4.3 |
| 12th month | 71.9 | 64.8 | 7.0 |

* To measure productivity loss, a survey was conducted to ascertain whether the patients were currently paid employees at each time point. The employment rates were presented for each group by time point to explain the result of considering the productivity costs of unemployed patients as zero from the sensitivity analysis results (Sensitivity analysis 3 in Appendix Table 3). Missing values were filled in using multiple imputations.
